# A Case Report of Rare and Lethal Methicillin-Resistant Staphylococcus aureus (MRSA) Peritonitis in Infancy

**DOI:** 10.7759/cureus.41303

**Published:** 2023-07-03

**Authors:** Shishir Kumar, Ratan Kumar, Kaushik Saha, Shivraj Chauhan, Kumar Diwakar

**Affiliations:** 1 Department of General Surgery, Tata Main Hospital, Jamshedpur, IND; 2 Pediatric Intensive Care Unit, Tata Main Hospital, Jamshedpur, IND; 3 Department of Pathology, Tata Main Hospital, Jamshedpur, IND; 4 Department of Pediatrics, Manipal Tata Medical College (MTMC) Tata Main Hospital, Jamshedpur, IND

**Keywords:** perforation peritonitis, appendicectomy, acute abdominal emergencies, methicillin resistant staphylococcus aureus (mrsa), primary peritonitis

## Abstract

Peritoneal inflammation without a discernible intraperitoneal source is referred to as primary peritonitis. Only 2% of pediatric acute abdominal crises are diagnosed preoperatively. Association with other infections is uncommon and is often limited to hepatic and urinary pathogens. Here, we describe a case of primary peritonitis in a one-month-old child who had laparotomy and appendicectomy as per the recommended treatment plan. There were no accompanying hepatic and urinary diseases. In this instance, methicillin-resistant Staphylococcus aureus (MRSA) was the responsible bacteria. The use of linezolid, as per the culture sensitivity report of intraperitoneal pus, ensured a smooth recovery in this case.

## Introduction

Primary peritonitis is defined as peritoneal inflammation in the absence of any identifiable intra-abdominal source [1]. Usually, this condition is associated with hepatic or urinary pathogens [2]. It is an uncommon disease and accounts for 2% of pediatric acute abdominal emergencies [3]. It is extremely difficult to diagnose preoperatively in neonates and infants. Standard workup is like any abdominal emergency for this age, viz ultrasound, X-ray, and, in some cases, CT scans if X-ray and ultrasound are inconclusive. Diagnosis is established if laparotomy rules out an intra-abdominal focus as a source of peritonitis [4]. In the case of localized collection, image-guided tap and culture-specific antibiotics are reasonable alternatives [5].

## Case presentation

**Figure 1:**
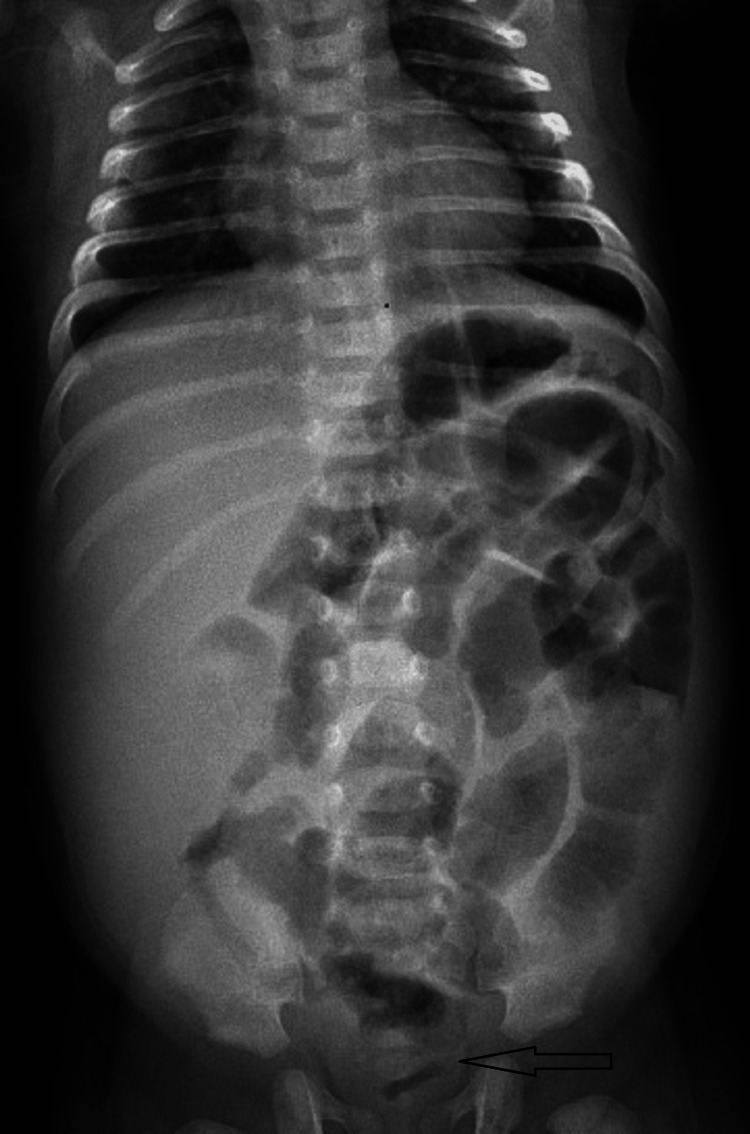
X-ray of the abdomen showing a gas shadow reaching the rectum and no pneumoperitoneum.

A 42-day-old boy (full term and normal vaginal delivery) was brought with complaints of abdominal distension and intermittent episodes of fever for 20 days. There was no history of obstipation, per rectal bleeding, or bilious vomiting. The baby was on exclusive breast feeds till the time of presentation. At the initial examination, the boy was normotensive for his age and had tachycardia (rate, 140 beats per minute) and tachypnoea (60 breaths per minute). Bowel sounds were sluggish. A digital rectal examination revealed semiformed stools. There was no history of delayed passage of meconium. Blood examination revealed raised C-reactive protein (CRP) (10 mg/dL), leucocytosis (17,000/mm^3^) with neutrophilia (74%). Platelet counts were normal (4.5 lacs/mm^3^). Urine microscopy results and liver function tests were normal. On palpation of the abdomen, there was tenderness in the right iliac fossa region. Abdominal X- ray was equivocal (Figure 1).

**Figure 2:**
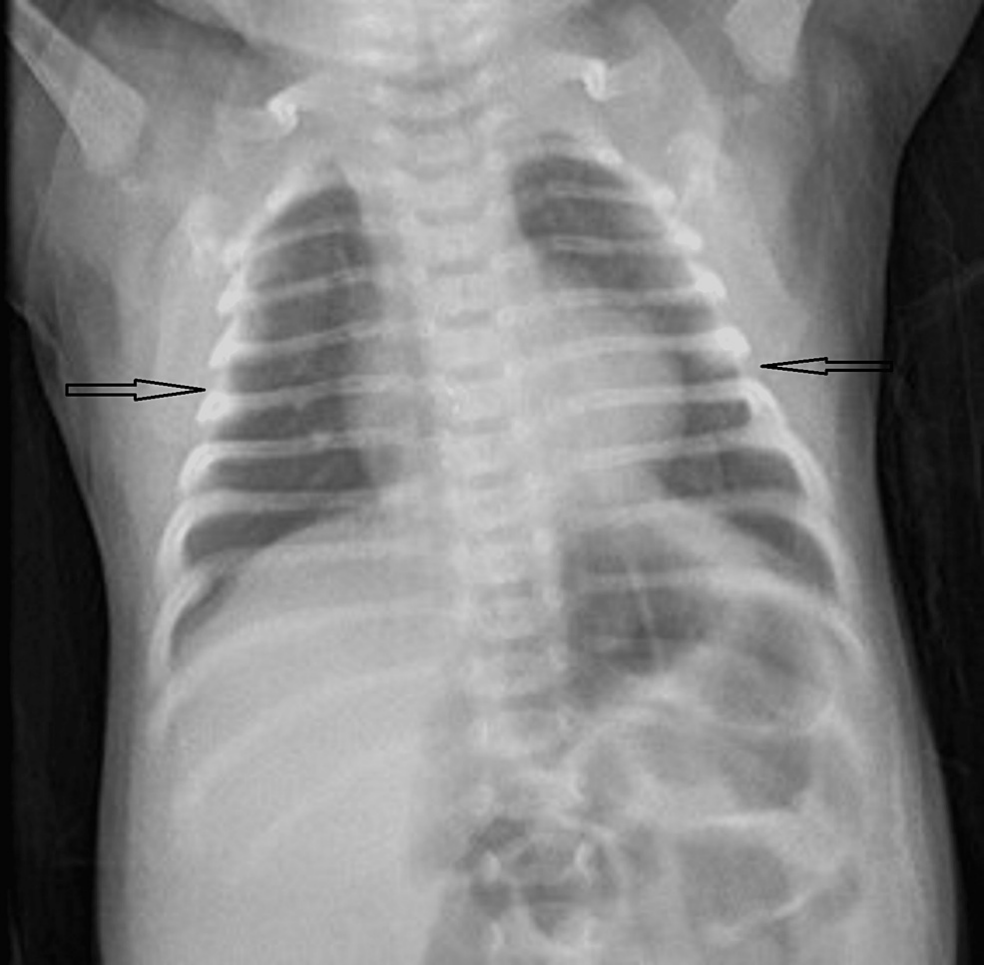
X-ray of the chest showing bilateral clear lung fields.

X-ray of the chest showed bilateral clear lung fields (Figure 2).

**Figure 3:**
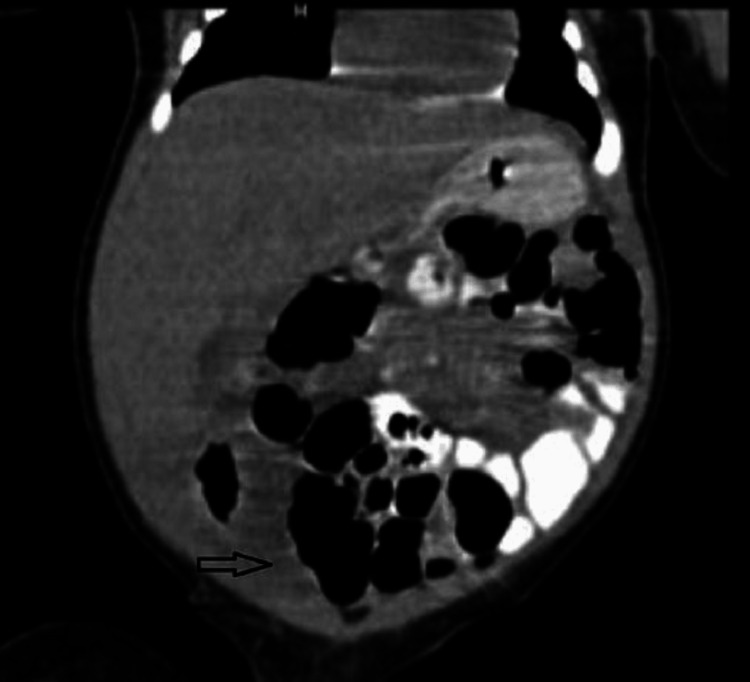
Contrast CT of the abdomen showing fluid in the right iliac fossa region (arrow). CT, computed tomography

**Figure 4:**
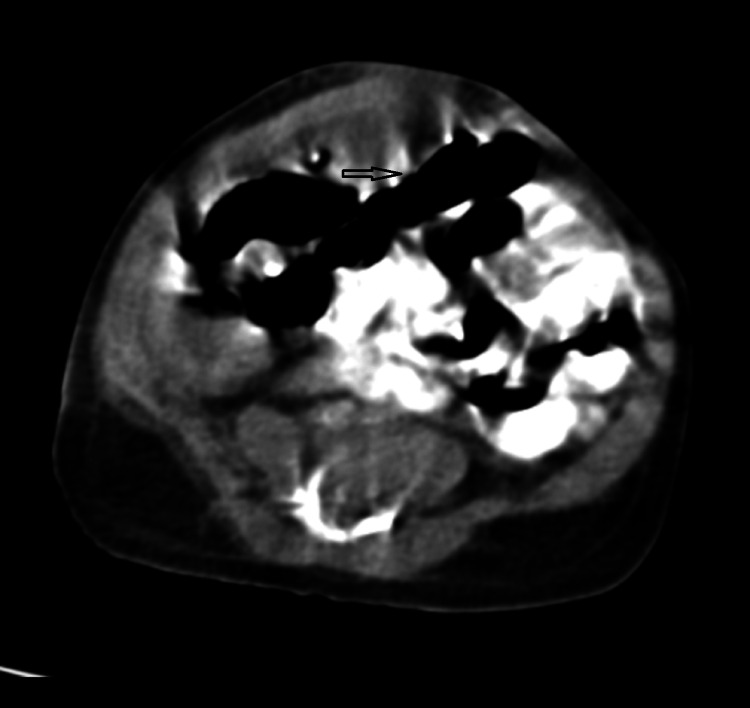
Contrast CT of the abdomen showing subtle peritoneal enhancement (arrow). CT, computed tomography

An ultrasonogram of the abdomen was non-contributory due to dilated bowel loops. Given diagnostic uncertainty, a contrast CT scan of the abdomen showed minimal hypodense collection in the right hypochondrium extending up to the right iliac fossa region (Figure 3) with peritoneal enhancement (Figure 4).

**Figure 5:**
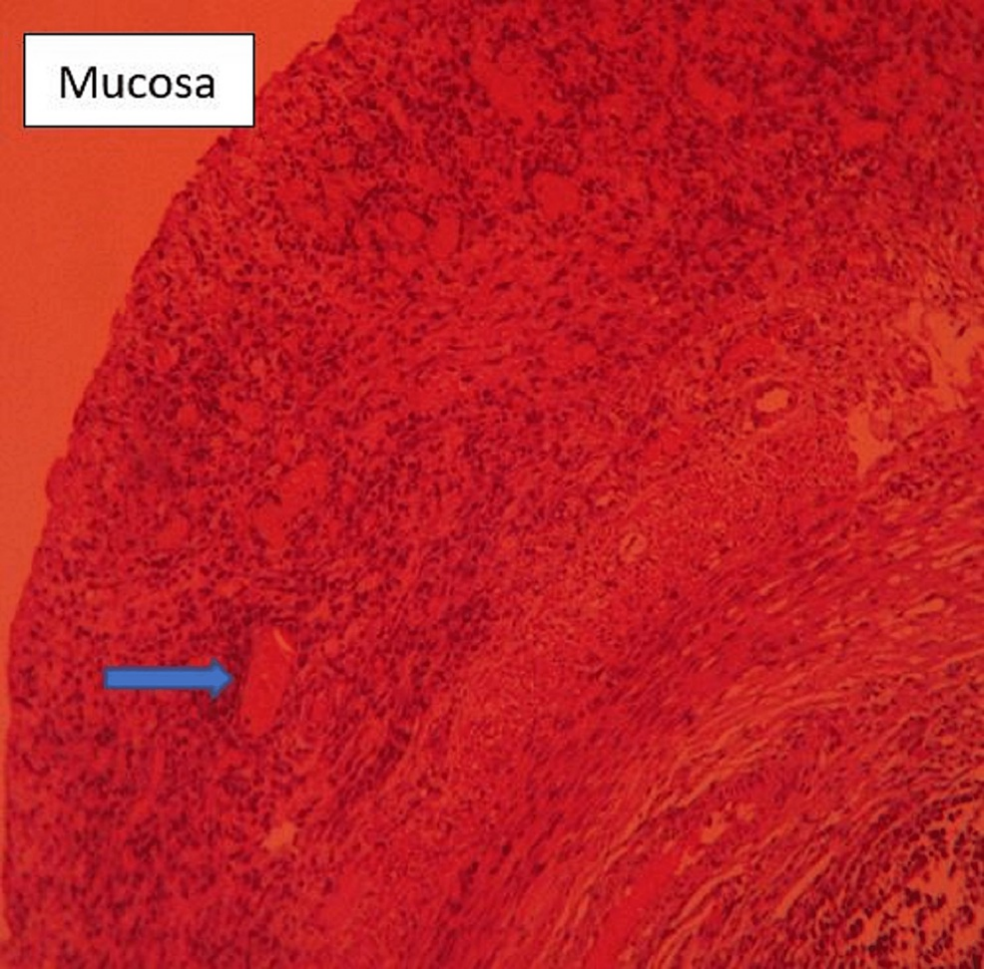
Normal mucosa with a lymphoid nodule (arrow) in the lamina (HE, 10× magnification). HE, hematoxylin and eosin

**Figure 6:**
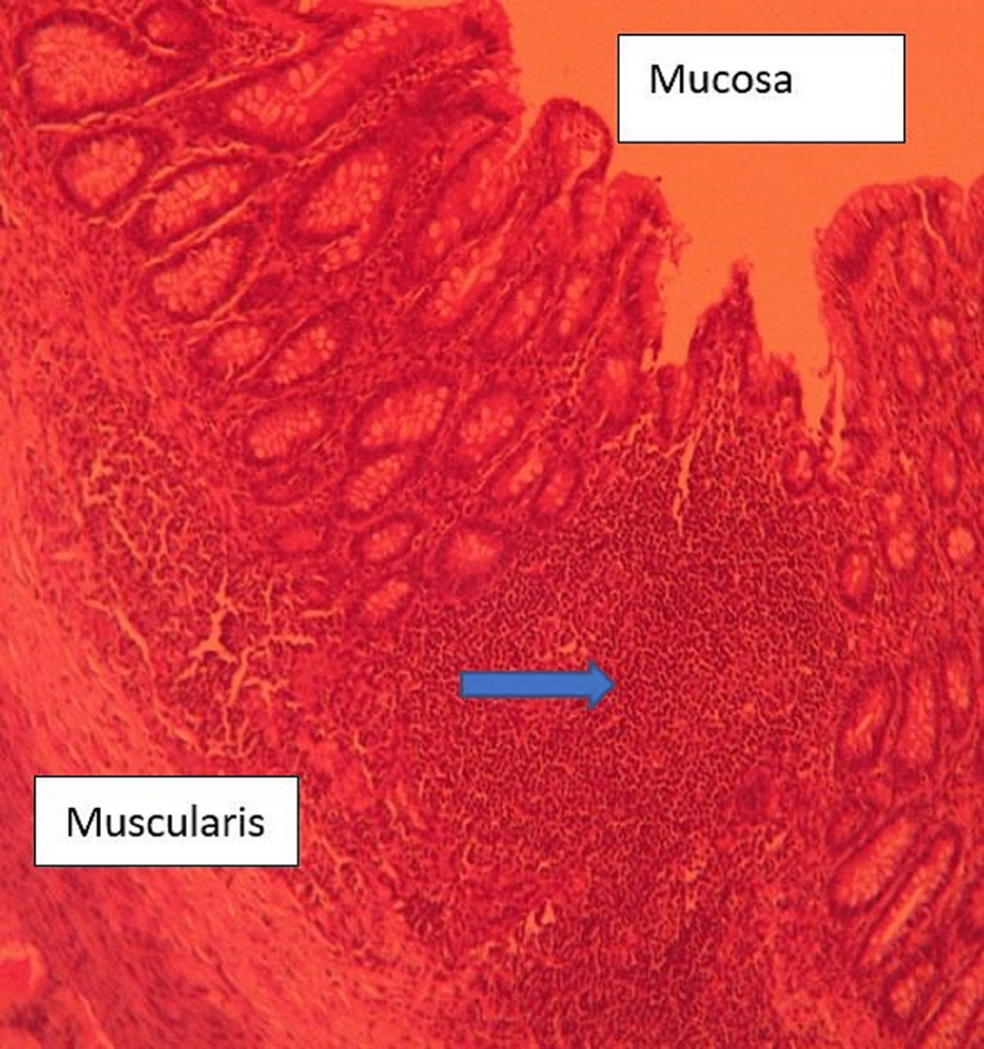
Higher magnification (40×) showing normal appendicular mucosa with cellular infiltration in the subserosa (arrow).

Given persistent abdominal distension, right iliac fossa tenderness, increased CRP and leucocytosis, and radiological evidence of peritoneal enhancement and free fluid, an exploratory laparotomy was undertaken which revealed pus collection in the right iliac fossa with pus flakes over serosa of the appendix. Peritoneal lavage with appendicectomy was done. The postoperative period was uneventful, with a resolution of hematological parameters by the second post operative day, and a resumption of full oral feeds by the third post operative day. Pus culture sensitivity was suggestive of methicillin-resistant Staphylococcus aureus (MRSA), sensitive to linezolid. Parenteral linezolid (10 mg/kg twice daily) for seven days, followed by oral linezolid for seven days ensured uneventful recovery. Histopathology of the resected appendix ruled out appendicitis as the cause of peritonitis (Figures 5-6).

## Discussion

Primary peritonitis is an uncommon disease accounting for 2% of patients presenting with an acute abdominal emergency. In clinical practice, it is difficult to differentiate from other causes of intra-abdominal sepsis, viz perforation peritonitis. In clinical practice, it is even more difficult to diagnose neonates and infants, in the absence of any hepatic or urinary pathologies. Even in this case, no hepatic or urological abnormalities could be identified. In neonates/infants with peritonitis, X-ray findings usually show the presence of free air or calcifications [6]. X-ray abdomen, in our case, was inconclusive. Even a CT scan of the abdomen showed only peritoneal enhancement, without any evidence of free air or collection.

The clinical course of primary peritonitis and perforation peritonitis usually are guiding factors in diagnosis and line of management [7]. Primary peritonitis usually develops under the clinical setting of an overwhelming infection. Perforation peritonitis is more acute in onset, with rapid clinical deterioration. The most common clinical associations of primary peritonitis are usually nephrotic syndrome, urinary tract infections, adrenogenital syndrome, and pneumonia [8]. The pathogens associated with peritoneal sepsis are usually gram-negative bacteria, mainly Escherichia coli or Klebsiella pneumoniae [9]. In this case, the diagnosis of primary peritonitis was made in the postoperative period after no intra-abdominal source of sepsis could be found, and urinary and liver tests came out normal. The perplexing fact was the isolation of MRSA in the intra-abdominal pus. In primary peritonitis, the main source of infection is usually blood, genitalia, transdiaphragmatic lymphatics, or transmural migration of gut bacteria [10]. In this case, blood and urinary cultures were sterile. There were no history or clinical findings suggestive of pneumonitis. Also, laparotomy did not reveal any signs of enteric or appendicular pathology. In literature, MRSA peritonitis has been described in association with the hematogenous dissemination of bacteria from an infected indwelling catheter, nasal carriers, skin lesions, or in the setting of peritoneal dialysis [11]. Treatment involves the eradication of carrier status, removal of the infected catheter, and prompt antibiotic therapy. In our case, the only conclusive evidence of MRSA was intra-abdominal pus, sensitive to linezolid.

The purpose behind reporting this case is to highlight the presence of MRSA in the abdomen without any antecedent history of indwelling catheterization, and its presence as only bacteria other than the usual suspects in the form of E. coli or Klebsiella in a case of primary peritonitis.

## Conclusions

Recovery is good in cases of primary peritonitis if culture-specific antibiotics are initiated, and etiology is established. Establishing a preoperative diagnosis of primary peritonitis in a one-month-old patient is difficult, especially in the absence of predisposing factors. In this case, undertaking laparotomy and doing peritoneal lavage and appendicectomy helped in source control (drainage of intra peritoneal pus pocket) and establishing the diagnosis by the negative histopathological report.
